# Electrically Switchable Film Structure of Conjugated Polymer Composites

**DOI:** 10.3390/ma15062219

**Published:** 2022-03-17

**Authors:** Kamil Awsiuk, Paweł Dąbczyński, Mateusz M. Marzec, Jakub Rysz, Ellen Moons, Andrzej Budkowski

**Affiliations:** 1 Marian Smoluchowski Institute of Physics, Jagiellonian University, Łojasiewicza 11, 30-348 Krakow, Poland; pawel.dabczynski@uj.edu.pl (P.D.); jakub.rysz@uj.edu.pl (J.R.); andrzej.budkowski@uj.edu.pl (A.B.); 2 Academic Centre for Materials and Nanotechnology, AGH University of Science and Technology, Mickiewicza 30, 30-059 Krakow, Poland; marzecm@agh.edu.pl; 3 Department of Engineering and Physics, Karlstad University, 65188 Karlstad, Sweden; ellen.moons@kau.se

**Keywords:** conjugated polymers, electric field, phase separation, poly(3-hexylthiophene), voltage-induced doping

## Abstract

Domains rich in different blend components phase-separate during deposition, creating a film morphology that determines the performance of active layers in organic electronics. However, morphological control either relies on additional fabrication steps or is limited to a small region where an external interaction is applied. Here, we show that different semiconductor-insulator polymer composites can be rapidly dip-coated with the film structure electrically switched between distinct morphologies during deposition guided by the meniscus formed between the stationary barrier and horizontally drawn solid substrate. Reversible and repeatable changes between the morphologies used in devices, e.g., lateral morphologies and stratified layers of semiconductors and insulators, or between phase-inverted droplet-like structures are manifested only for one polarity of the voltage applied across the meniscus as a rectangular pulse. This phenomenon points to a novel mechanism, related to voltage-induced doping and the doping-dependent solubility of the conjugated polymer, equivalent to an increased semiconductor content that controls the composite morphologies. This is effective only for the positively polarized substrate rather than the barrier, as the former entrains the nearby lower part of the coating solution that forms the final composite film. The mechanism, applied to the pristine semiconductor solution, results in an increased semiconductor deposition and 40-times higher film conductance.

## 1. Introduction

The solution-coating of polymer blends provides a one-step deposition of different components, usually accompanied by their phase separation [1]. This processing is key to producing electronics at low cost on large areas and is based on semiconducting conjugated polymers dissolved and mixed in organic solvents with other functional species [2]. Phase-separated domains can self-assemble into film structures, with at least one domain type forming a continuous phase. Different morphologies of spin-coated polymer composites have found suitable applications: light-emitting diodes (LEDs) based on vertically separated layers of an electron donor and an electron acceptor are superior to those using laterally assembled domains [3]. Films with extended donor–acceptor interfaces are imperative for photodiodes and solar cells (SCs) [4], with the device performance enhanced by vertical separation [5]. Insulating matrices with lateral droplets of semiconducting polythiophene are used in LEDs with voltage-controlled color [6]. Self-stratified bilayers of polythiophene semiconductors and gate dielectrics [7] or insulating encapsulants prolonging the operational lifetime [8] are applied in field-effect transistors (FETs). In addition, such bilayers exhibit an extraordinary conduction increase after the deposition of d-block metal complexes [9]. Finally, lateral [10,11] and vertical [12] phase separation, which is selective on regularly designated substrate areas, enhances the performance of LEDs [10] and SCs [11] and simplifies the production of FET arrays [12].

For emerging applications, suitable adjustment of the film morphology based on an improved understanding of structure formation during polymer blend deposition is desirable. This formation is a rapid and complex nonequilibrium process sensitive to many parameters. By setting these parameters prior to coating, morphological control can be obtained. For spin-coated semiconductor-insulator composites, methods to switch between lateral and vertical film structures involving changes in the blend composition, homologous component, solvent, or substrate chemistry have been demonstrated [12,13,14]. In the search for other external interactions [15], an electric field has been considered [16,17], but the modified structure of solvent-cast blends was restricted to the region between electrodes [18,19]. To date, no coating approach without either additional fabrication steps or small-area limitations that produce polymer composite films with reversible and repeatable changes between distinct morphologies has been achieved.

**Figure 1:**
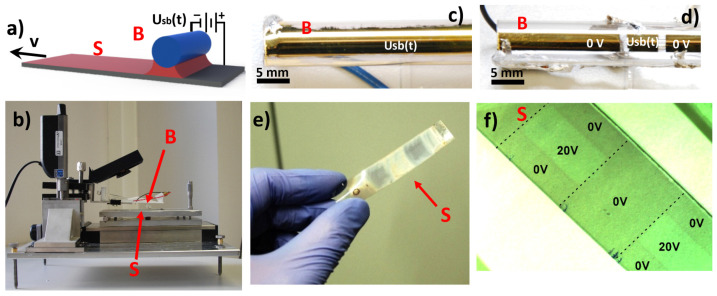
(**a**) Sketch and (**b**) photo of home-built device for the horizontal-dip coating process of a conductive polymer composite with an electrically modified film structure. An electric field is provided by a pulsed voltage *Usb*(*t*) applied between (**c**) one central or (**d**) multiple separated metallic electrode(s) on the glass coating barrier (B) and the conductive substrate (S). (**e**) Electrically modified film structure of the semiconductor-insulator polymer composite (RP3HT/PEG-PCL) on a flexible substrate with coating barrier (**c**), indicating the processability in large-area fabrication. (**f**) Modified film structure of the RP3HT/PEG-PCL composite on rigid ITO-coated glass slide fabricated with the coating barrier with multiple electrodes (**d**).

Novel high-throughput coating techniques, compatible with roll-to-roll fabrication, rely on withdrawing the substrate through the solution meniscus [20,21,22]. They offer a little studied option to modify the morphology along the deposited blend film by temporal variation in the coating process [23]. To pursue this approach, we introduce and vary an electric field across the meniscus-dominated region of the solution (Figure 1a). The direct purpose of this work is to demonstrate such a methodology for semiconductor-insulator polymer blends, deposited from the solution onto a rapidly drawn horizontal substrates, and to examine the mechanism of the electrically controlled morphology, formed along the deposited films by phase-separated blend domains. To evidence this effect on both rigid and flexible horizontal substrates, a novel device for electrically modified horizonal-dip coating is introduced (Section 3.1). The generality of the effect is examined for three binary blends of two polythiophene semiconductors and two polymer insulators (Section 3.2). To determine the relevant mechanism, the impact of the electric field on the film structure formation is scrutinized considering existing literature (Section 3.3) and new experiments (Section 3.4 and Section 3.5). They show that the dominant driving force behind the morphology control is not related to electro-kinetic effects (Section 3.4) but rather to the electrically modified doping and the solubility of polymer semiconductors (Section 3.5). 

The main novelty of this work is the concept of the electrically modified temporal variation in the coating process that controls the local polymer blend film morphology. In turn, the potential of the introduced methodology for all meniscus-guided coating techniques, e.g., used in the fabrication of large-area organic electronics, accounts for the importance of this study.

The method presented here of the electrically regulated lateral control and switch of film structures is active already during the solution coating of semiconductor polymer blends. In contrast, an additional step of substrate pattering, e.g., by lithography, that precedes the blend coating was previously needed to secure the spatial control of phase-separated domains [13]. As such domains form the elements with complementary functions [24] for electronics and, potentially, for biotechnology, the fabrication of various polymer-based devices can be simplified. The manufacture of the arrays of LED pixels [10] and SCs [11] or protein microarrays [25] (possibly, with conjugated polymers [26]) might benefit from the spatial control of lateral morphologies. In turn, construction of the assemblages of FETs [12,27] (potentially, FET-based biosensors [28,29] or light-detection position sensors [30]) can benefit from lateral control of the vertically separated lamellar structures.

## 2. Materials and Methods

### 2.1. Materials

Regioregular poly(3-hexylthiophene-2,5-diyl) (RP3HT, Mw~15,000–45,000, head-to-tail regioregularity: >95%, electronic grade) was purchased from Sigma Aldrich, and poly(3,3’’’-didodecyl quarter thiophene) (PQT12, Mw~15,000–30,000) was supplied by American Dye Source. Insulating polymers, poly(ethylene glycol)-block-poly(ε−caprolactone) methyl ether (PCL Mn~15,000, PEG Mn~5000) and poly(methyl methacrylate) (PMMA, Mw = 65,000, Mw/Mn = 1.05), were sourced from Sigma Aldrich and Polymer Standard Service GmbH, respectively. As a common solvent, chlorobenzene purchased from POCH S.A. was used.

### 2.2. Substrates

Indium tin oxide (ITO)-coated polished float glass (Rs = 8–12 ohms, purchased from Delta Technologies, Loveland, CO, USA), with a SiO_2_ passivation layer applied directly on the glass prior to ITO coating, was used as a rigid substrate for the deposition of conductive polymers and their composites. The size of the substrate was approximately 75 × 13 mm^2^. In turn, as a flexible substrate, polyethylene terephthalate (PET) foil coated by ITO (Rs = 60 ohms, purchased from Sigma Aldrich, Darmstadt, Germany) was applied. The current–voltage characteristics of the fabricated polymer thin film was evaluated using the ITO Glass Scale-Up PV and OLED Substrate (purchased from Ossila, Sheffield, UK). Prior to polymer film formation, glass substrates with an ITO layer were cleaned by sonification in chlorobenzene for 5 min, and dried with N_2_ steam, whereas PET foil coated by ITO was cleaned with isopropyl alcohol.

### 2.3. Films Prepared by Electrically Controlled Horizontal-Dip (H-Dip) Coating

Three pairs of semiconductors and insulators, RP3HT/PEG-PCL, RP3HT/PMMA, and PQT12/PEG-PCL, were dissolved with different weight ratios (w:w) in chlorobenzene. The blends of RP3HT/PEG-PCL (60:40, 55:45, 50:50, and 45:55), RP3HT/PMMA (60:40 and 55:45), and PQT12/PEG-PCL (50:50) were prepared at a fixed total polymer concentration of 15 mg mL^−1^. The same concentration was used to dissolve pristine RP3HT. All solutions were kept overnight at 60 °C prior to deposition. Films of conductive polymer composites and pristine RP3HT were prepared by electrically controlled horizontal-dip (H-dip) coating using the home-built device depicted in Figure 1b. First, the height was set to ~500 µm, and the tilt of the barrier (B) in relation to the substrate (S) was adjusted and maintained during the coating. Then, a small amount of solution was placed between the barrier and substrate near the edge of the latter, and the substrate was moved with a velocity *v* of 1 mm/s or 2 mm/s using a computer-controlled linear stage (Newport UTS100). Finally, during the meniscus-guided deposition of a polymer film, an electric potential difference *Usb* between the substrate and barrier was introduced. The voltage *Usb* was alternated with a typical switching time of 6 s (for *v* = 2 mm/s) or 12 s (for *v* = 1 mm/s) following a two-level (0, Umax) or a three-level (0, Umax, 0, −Umax) sequence, with a maximum voltage |Umax| of 20 V or 30 V, to form on the substrate at least four composite regions, each with a typical size of approximately 12 × 13 mm^2^. For H-dip coating experiments with pristine RP3HT, a three-level (0, Umax, 0, −Umax) sequence of 30 V and asymmetric switching times were applied. All samples were prepared under ambient conditions.

### 2.4. Microscopic Characterization

Optical and fluorescence micrographs were acquired with an Olympus BX 51 reflection microscope equipped with a halogen lamp, a U-MNG2 fluorescence mirror unit (excitation filter at 530–550 nm and a 590 nm low-pass filter), and a digital camera (DP72).

### 2.5. AFM Examination

An Agilent 5500 scanning probe microscope working in contact mode was used to examine the topography of polymer films. AFM micrographs were analyzed with PicoImage software (Agilent, Santa Clara, CA, US).

### 2.6. ToF–SIMS Characterization

The composition of polymer films was examined with a TOF.SIMS 5 (ION-TOF GmbH, Münster, Germany) instrument, a secondary ion mass spectrometer with a time-of-flight analyzer equipped with a 30 keV bismuth liquid metal ion gun. Surface chemical maps of composite component distributions were acquired in imaging mode, applying Bi_3_^+^ clusters (primary ions) with an ion dose density of less than 10^12^ ion/cm^2^, to ensure static mode conditions. To determine the 3D distributions of the composite components in the polymer films, the TOF.SIMS 5 system working in dual-beam mode was used. To obtain the 3D distributions, the samples were sputtered with a C_60_ ion beam (20 keV eV, 1 nA) rastered over a 700 µm × 700 µm area, exposing the deeper layers of the film. The structures revealed in the crater center were analyzed with a focused bismuth beam (Bi_3_^+^, imaging mode) rastered over a 20 µm × 20 µm area. Sulfur-containing ions (e.g., HS^−^, C_3_H_4_S^−^, C_2_S^−^, C_6_HS^−^, and C_7_H_5_S^−^) and oxygen-containing ions (e.g., O^−^, CHO_2_^−^, C_2_HO^−^, C_2_H_2_O^−^, C_2_H_3_O^−^, and C_3_H_3_O_2_^−^) were chosen to trace the RP3HT and PEG-PCL components, respectively. 

### 2.7. Ellipsometry Measurements

A Sentech SE800 spectroscopic ellipsometer, equipped with a microspot accessory, was used to determine the optical properties (e.g., extinction and absorption coefficient spectra) and film thickness of the deposited semiconducting polymer (RP3HT).

### 2.8. Voltage Variation in the Course of H-Dip Coating

Spatial variation in the voltage *Usb* as a function of the position along the RP3HT film was imposed with a computer-controlled Keithley 2400 source-meter unit and a linear stage controller.

### 2.9. Electrical Characterization

Current–voltage characteristics of pristine RP3HT film were measured in a self-made measuring system. To this end, a RP3HT thin film was fabricated on scale-up pre-patterned ITO substrates S271 provided by Ossila. During film formation, the finger electrodes were grounded, whereas the *Usb*(*t*) voltage was applied to the barrier. After film fabrication, the samples were closed in the measuring chamber under an argon atmosphere and current–voltage characteristics were collected by the measuring system working with a computer-controlled Keithley 2400 source-meter unit.

## 3. Results and Discussion

### 3.1. Electrically Modified Horizontal-Dip Coating

In the first set of experiments, we examined whether the composite structure formed during meniscus-guided deposition onto a rapidly drawn solid substrate [22] could be modified along the deposited film by temporally varying the applied E-field. To this end, we studied the horizontal-dip coating process outlined in Figure 1a using the device presented in Figure 1b. In this configuration, a solid substrate (S) is moved horizontally (at speed *v*) with respect to the stationary cylindrical barrier (B). After coating solution application, a meniscus is formed in the gap between the substrate and the barrier, exerting a Laplace pressure that reduces the final film thickness to the tens-of-nanometers scale, with the exact value related to the coating speed *v* by Landau–Levich theory [20]. In our presented device, an electric field (E-field) perpendicular to the substrate is introduced into the capillarity-dominated region of the coating solution (Figure 1a). This field is provided by a voltage *Usb*(*t*) that can be varied with time, applied between the one central or multiple separated metallic (Au) electrode(s) fabricated on the glass barrier (Figure 1c,d) and the conductive substrate (ITO on glass or PET foil, Figure 1e,f). Additionally, a constant height of the barrier (~500 µm, with micrometer accuracy) and its parallel alignment with respect to the substrate are maintained thanks to our developed extended AFM-based control system (Figure S1). 

As a model composite, we used a semiconductor, regioregular poly(3-hexylthiophene-2,5-diyl) (RP3HT), blended in chlorobenzene with an insulator, poly(ethylene glycol)-block-poly(ε-caprolactone) methyl ether (PEG-PCL), keeping the 1:1 RP3HT/PEG-PLC weight ratio. The results of horizontal-dip coating combined with the E-field that was periodically switched on and off (with *Usb*(*t*) alternating between 0 and 20 V) are presented in Figure 1e,f. These results show that the composite structure can be electrically modified, as indicated by its variation along the films deposited on both substrates, indicating the processability in large-area fabrication. In addition, an exchange of the one central electrode for multiple electrodes on barrier B (Figure 1c) provides another method, in addition to the varied voltage *Usb*(*t*), for substrate patterning with structurally different regions (Figure 1f).

### 3.2. Film Morphology Switched by an Electric Field (E-Field)

**Figure 2:**
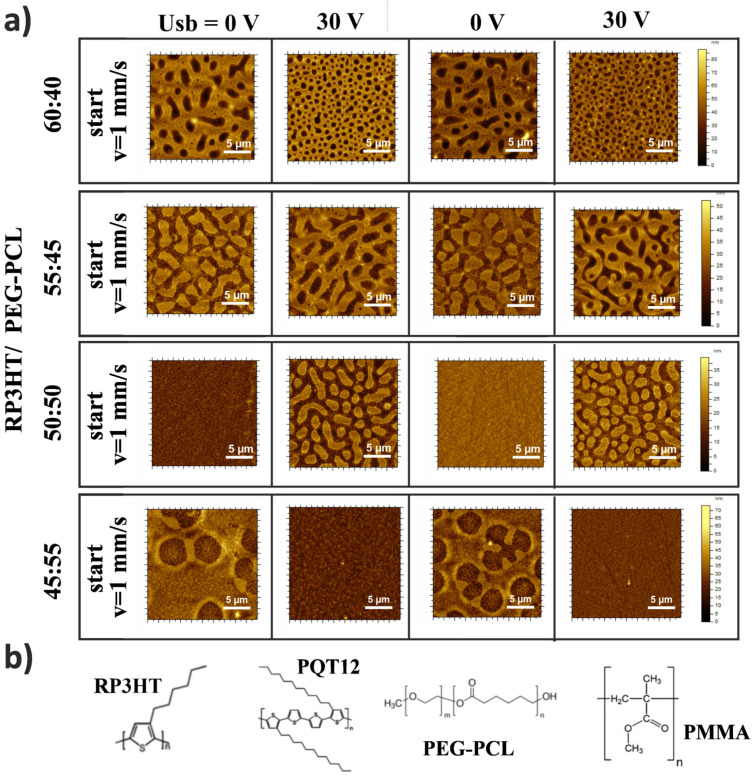
Reversible and repeatable electrically switched changes between different morphologies along the cast film. (**a**) AFM height images of RP3HT/PEG-PCL composites dissolved with different weight ratios (rows) in chlorobenzene and H-dip-coated with a pulsed E-field provided by a 30 V voltage *Usb*(*t*) periodically switched on and off (columns). (**b**) Chemical structures of the semiconductors, RP3HT and PQT12, and insulators, PEG-PCL and PMMA, that form the bicomponent polymer composites examined in this study, all dissolved in chlorobenzene at a fixed total polymer concentration of 1.33 wt%.

To explore the electrical modification of the film structure in detail, H-dip coating experiments were performed for a series of model composites RP3HT/PEG-PCL with different weight ratios (the rows in Figure 2a). The regions along the composite films formed with a pulsed 30 V voltage *Usb* and the E-field periodically switched off and on (the columns in Figure 2a) are depicted by AFM height micrographs taken along the cast film. The AFM images reflect the phase-separated domains as the height contrast differentiates different phases of the RP3HT composites [14], as confirmed by chemical ToF–SIMS imaging (Figures S2 and S3). In particular, elevated domains rich in RP3HT and depressed domains rich in PEG-PCL are concluded for the lateral morphologies in Figure 2. Similarly, stratified layers rich in RP3HT and facing air are deduced for the lamellar structures.

The results of Figure 2 are extremely beneficial, as they show that the proposed approach enables the coating of the composites with reversible and repeatable changes between the distinct film morphologies, electrically switched at a frequency compatible with the rapid speed deposition. Different pairs of interchanged composite morphologies can be obtained, depending on the component weight ratio. In particular, E-field-induced changes can occur between the phase-inverted droplet-like lateral structures (the second row) as well as between the lateral and lamellar morphologies (the third row).

To examine the generality of the above phenomenon, we also studied two other bicomponent composites containing RP3HT and PEG-PCL, namely, RP3HT/PMMA and PQT12/PEG-PCL. Overall, we examined three pairs of semiconductors, RP3HT or poly(3,3‴-didodecyl quarter thiophene) (PQT12), and insulators, PEG-PCL or poly(methyl methacrylate) (PMMA). The RP3HT [4,7,9,11,13,20,22,24,30] and PQT12 [8,12,23] polymers were chosen for this study, as representatives of the polythiophenes, a heavily studied family of processable, semiconducting polymers [31]. While RP3HT is the most commonly used material in plastic electronics, PQT12 has an enhanced resistance to doping degradation caused by air [31]. Both polymer insulators [25,32,33] and semiconductors [26,28,29] used in these studies are biocompatible, and they were used for the fabrication of protein micro-arrays [25,33] or FET-based biosensors [28,29]. In addition, similar composites as in our studies were used for electrospinning (RP3HT/PCL) [34] and the facile manufacture of FETs (RP3HT/PMMA) [7,35] or FET arrays (PQT12/PMMA) [12].

The results of the additional H-dip coating experiments show that reversible and repeatable changes between distinct film morphologies can be electrically induced for different conductive polymer composites, including RP3HT/PMMA (Figures S4a and S5) and PQT12/PEG-PCL (Figure S4b). For RP3HT/PMMA, we also analyzed the impact of both the composite composition and coating speed. As previously described, the component weight ratio determines the pair of electrically interchanged composite morphologies. For the 55:45 RP3HT/PMMA composition, the semiconductor dispersed in the insulator is phase-inverted into insulator domains in the semiconductor and vice versa (Figure S4a). The size of the phase-separated domains depends on the coating speed, as observed recently [23].

### 3.3. E-Field Impact on Film Structure Formation

The results of Figure 2 and Figure S4 are surprising considering the existing literature. First, the E-field between the substrate and the barrier starts to disappear for a given substrate section when it leaves the meniscus region of the coating solution (see Figure 1a). This moment also initiates enhanced solvent removal by evaporation, leading to the microscopically observed onset of phase demixing [36]. However, although the electrically induced modifications are effective only at the very beginning of film structure formation, they have a profound effect on the final film morphology. Second, the pronounced morphological changes are induced by very short exposures of the semiconductor-insulator composites to moderate E-fields (up to ~12 s at 400–600 V/cm). In contrast, visible modifications of the phase domain structure were previously reported for much longer exposures and stronger E-fields (ca. 20–30 min at 4–12 kV/cm), applied during solvent-casting of insulator–insulator polymer blends [19,37,38]. Our observations correspond more to the morphology changes reported for a moderate E-field introduced to the conjugated polymer composite RP3HT/PMMA shortly after spin-coating but prior to complete solvent evaporation [39].

Other features of the electrical effects are manifested with respect to film structure formation in the absence of an E-field. Phase demixing, triggered by the volatilizing solvent, is followed by a complex nonequilibrium structure evolution until the transition into a solid. This phenomenon results in a layered morphology at one extreme and a lateral phase arrangement at the other [2,40]. While lamellar structures have been related to surface-directed phase separation [13,14,41], the mechanisms of lateral phase separation are not completely resolved [2,40]. Lateral structures can be formed by the break-up of the bilayers through interfacial instability [40,42]. This instability also accounts for the switch between the final lateral and vertical structures of the blend film series [43]. Lateral domain arrangement can also be a result of quasi-2D coarsening [36]. The final film morphology is specified by the blend composition [2,7,44,45]. In the above context, two comments on our observations can be made. First, the E-field can induce a morphology switch (Figure 2a) not only from but also into a lamellar structure (cf. 50:50 and 45:55 RP3HT/PEG-PCL). Therefore, electrostatic pressure, known to drive interfacial instability [16], is not a decisive force here. Second, the composition series of morphologies obtained in the presence and absence of an E-field are related. More specifically, the electrically modified film structures of RP3HT/PEG-PCL and RP3HT/PMMA correspond well to those obtained without an E-field but with the RP3HT content higher by ~5 wt% (cf. the 30 V and 0 V columns in Figure 2a and Figure S4a). This result extends the relation between the morphology and composition with the E-field effect, which is equivalent to an increase in the semiconductor content. This unique feature will be revisited when examining possible mechanisms of electrical modifications in film structure formation.

We note also that for any bicomponent composite (with given component weight ratio), the same morphology is obtained under voltage-off conditions prior to and after the voltage pulse (the first and the third column in Figure 2 and Figure S4). This reflects the absence of any chemical degradation of polymer components that would modify phase demixing. In addition, auxiliary ToF–SIMS measurements show that the thiophene composition, modified during degradation [46], is unaffected by a voltage pulse (Figure S6). This is in accordance with the recent report of photoelectron spectroscopy studies on polythiophene degradation caused by pulsed voltage, observed only after very long (~5 h) cycling in an electrolyte rather than in a solvent [46]. In contrast to the results of short processing from solution (*v* = 1 or 2 mm/s), used here, conventional melt processing of RP3HT at elevated temperatures would result (after just 10 min at 250 °C) in substantial chemical degradation [47]. This conclusion holds also for insulating polymers, PEG-PCL [48] or PMMA [49].

### 3.4. Morphologies Formed in Reversed and Zero E-Fields

To understand the origins of electrical control of the composite morphology, we next analyzed the effects of a reversed E-field. Generally, two classes of electro-kinetic forces are relevant for conjugated polymers suspended in a solvent: Electrophoresis acts on charged, partially doped molecules, with a reversed motion induced by a reversed E-field orientation [50]. In turn, dielectrophoresis of polarizable, even uncharged, molecules results in motion driven by an inhomogeneous field, i.e., by the gradient of E^2^ [50]. Other mechanisms dependent on E^2^ are also insensitive to the E-field orientation: A modified energy of mixing leads to a changed phase diagram [51]. The interfacial pressure between phases with dielectric constant mismatch results in dispersed phase deformation [18], morphological variation [38], and interfacial instability [16].

**Figure 3:**
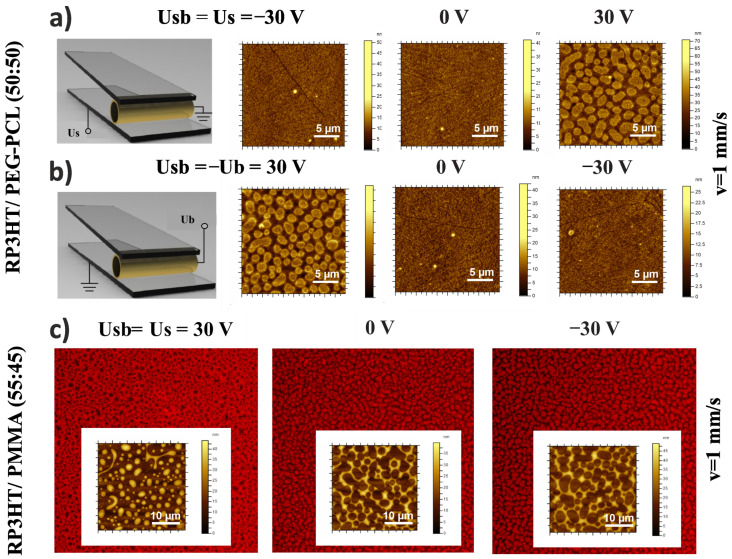
Impact of electric field orientation on the film morphology of H-dip-coated composites: (**a**,**b**) RP3HT/PEG-PCL (50:50 by weight) and (**c**) RP3HT/PMMA (55:45) electrically switched between the lamellar and lateral structures (**a**,**b**) and between the phase-inverted droplet-like structures (**c**). AFM height images of the composites coated with the E-field changed between the opposite orientations, with a zero field interval between both regimes, controlled by the voltage *Usb*(*t*) applied between the substrate and barrier. Equivalent results are obtained for the same *Usb* values, irrespective of the zero potential being connected to either the barrier (**a**) or the substrate (**b**). (**c**) Fluorescence micrographs of the RP3HT-rich phase revealing the AFM height contrast between the phase-separated domains of RP3HT/PMMA.

**Figure 4:**
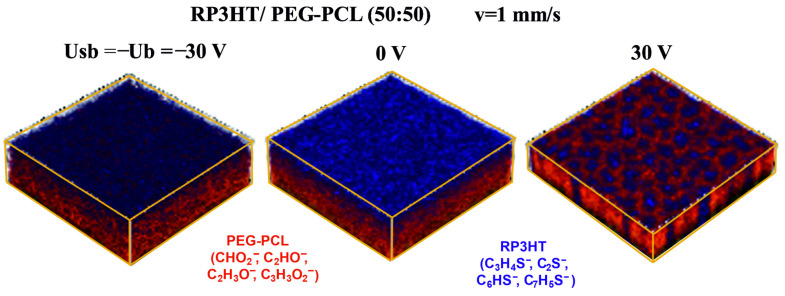
Effect of E-field orientation on the component distribution in the H-dip-coated film of the binary composite RP3HT/PEG-PCL (50:50), with the morphology switched between overall lamellar and lateral structures. Spatial distribution of composite components (RP3HT, blue and PEG-PCL, red), determined with 3D ToF–SIMS for the composite coated with the E-field changed between the opposite orientations, with a zero field interval between both regimes, controlled by the voltage *Usb*(*t*) between the substrate and barrier. The depth from the original surface is measured based on the sputtering time.

The impact of E-field orientation on film morphology was analyzed for two H-dip-coated composites RP3HT/PEG-PCL (Figure 3a,b and Figure 4) and RP3HT/PMMA (Figure 3c). Their compositions correspond to the electrically induced changes between the lamellar and lateral structures (cf. the third row of Figure 2a) and between the phase-inverted droplet-like structures (cf. the second row of Figure S4a). The results, presented as AFM height images (Figure 3), fluorescence micrographs (Figure 3c), and 3D ToF–SIMS chemical distributions (Figure 4), are revealing. First, irrespective of the electric potential being applied to either the substrate (Figure 3a) or the coating barrier (Figure 3b), with the other electrode grounded, the morphologies obtained for a given potential difference *Usb* are similar. This result underlines the main role played here by the meniscus region between the substrate and the barrier, rather than the downstream substrate region with macroscopic onset of phase separation [36]. Second, even more importantly, the E-fields with reversed orientation (*Usb* = 30 V vs. −30 V) have different impacts on the morphology (Figure 3 and Figure 4). Therefore, dielectrophoresis and other mechanisms dependent on E^2^ [16,18,38,50,51] fail to explain this work’s observations. Third, a negative *Usb* value does not cause any visible changes, either in the composite morphology (Figure 3) or in the component distribution in the film (Figure 4), compared to the zero-field case. Hence, electrophoresis cannot be the dominant driving force here.

### 3.5. Electrically Controlled Polythiophene Solubility

The results presented above indicate that the observed electrical modification of the composite film morphology must be based on a novel mechanism. Its essential features, revealed by the experiments, include relevance to solutions containing conjugated polymers, induction by short exposures to small potential differences *Usb* (moderate E-fields), and effects equivalent to an increased semiconductor content on the coated substrate. The specific properties of this mechanism include operation in the meniscus between the stationary barrier and entrained substrate, termination prior to the macroscopic onset of phase separation, and nonzero effects observed only for the substrate positively polarized with respect to the barrier.

The essential features of our observations can be related to doping-induced solubility control of conjugated polymers [52]. The solubility of polythiophene films in organic solvents can be rapidly (<1 s) “switched off” by molecules that induce p-type doping (oxidation) and recovered by chemical or optical de-doping [52,53]. Hole addition to polythiophenes in films and solutions shifts the equilibrium between solvated and aggregated states in favor of the aggregates [52]. Hole injection into the polymers can be realized [54] by not only (opto)chemical but also electrochemical [55,56] and interfacial [50,55] doping, with the latter two observed for polythiophene films positively polarized by small potential differences [50,55,56]. Additionally, polythiophenes in solutions can be rapidly and reversibly oxidized (within seconds) by a varied electric potential [57]. Therefore, electrically switched doping was observed to reversibly control the polythiophene solubility, i.e., its precipitation and solubilization [57].

**Figure 5:**
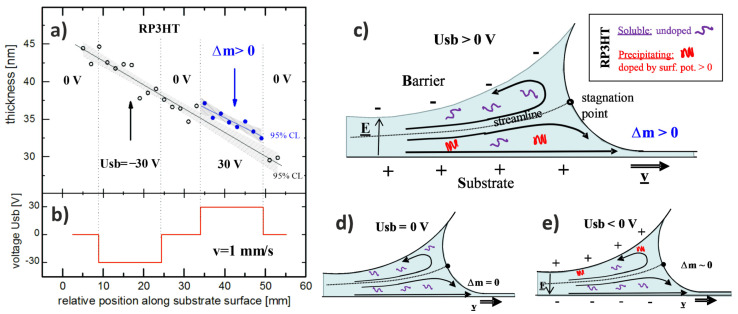
Electrically controlled content of RP3HT in the solution-coated films based on a novel mechanism involving (**a**,**b**) voltage-controlled RP3HT solubility and (**c**–**e**) flow pattern asymmetry between the stationary barrier and moving substrate during the H-dip coating process. The film thickness of a pristine RP3HT layer (**a**), determined after H-dip coating (from chlorobenzene, 15 mg/mL, *v* = 1 mm/s), and the voltage *Usb*(*t*) (**b**) imposed between the substrate and barrier in the course of coating are plotted as a function of the position along the layer. The extents of nonzero voltages are indicated by the vertical dotted lines. (**c**–**e**) Qualitative sketch of streamlines, typical for dip coating, in which the coating solution layer is split into two around a stagnation point, with the lower part entrained by the substrate and the upper one returning along the barrier to the reservoir in the meniscus. This situation introduces an asymmetry (with respect to *Usb*) to the solubility control and hence to the RP3HT content variation in the coated composites.

To confirm such a scenario for solution coating, we examined a pristine RP3HT film H-dip-coated with a varied three-level (0 V, −30 V, 0 V, 30 V) voltage *Usb*(*t*) applied. Ellipsometry spectra of the extinction coefficient, determined along the film, revealed features corroborating the existence of aggregates (Figure S7), supporting the above model. Next, the variation in the film thickness with position along the RP3HT film was determined with ellipsometry (Figure 5a), with a similar variation in the voltage *Usb*(*t*) (Figure 5b) imposed during the coating experiment. As a result, the complete regions with nonzero voltages applied were marked. These data present the main finding, supporting the electrically controlled solubility of RP3HT. Namely, despite a linear decay of the film thickness (Figure 5a), much stronger for RP3HT films than for its composites, an increased RP3HT deposition (Δ*m* > 0) is manifested for the region with positive voltage *Usb* applied (cf. the regimes with *Usb* = 30 V and *Usb* ≠ 30 V). Apparently, this phenomenon cannot be prevented by electrostatic interactions between the aggregates, with holes delocalized along their chains [52], and the positively charged substrate, similar to the deposition of charged macromolecules [58]. The additional electrically induced increase in the RP3HT film thickness (ca. 2 nm above 33 nm, averaged for *Usb* = 30 V) is equivalent to an increase in the RP3HT loading on the substrate by ~6 wt%. This result accords well with the conclusion on the electrically modified composite morphologies, in which the E-field effect is equivalent to an increase in RP3HT content by ~5 wt%.

**Figure 6:**
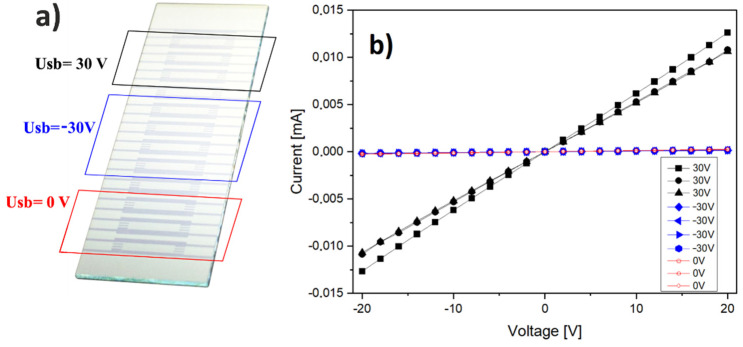
Electrically modified polymer deposition in the solution-coated pristine RP3HT film is accompanied by changed charge transport properties. (**a**) A scale-up substrate with 12 sets of ITO finger electrodes. The color frames mark film regions with different voltages *Usb* applied during H-dip coating. (**b**) Current–voltage characteristics of the electric circuits for the RP3HT film sections formed at different voltages *Usb*. Note that *Usb* = 30 V induces both a higher conductance and increased RP3HT deposition (Δ*m* > 0 in Figure 5a).

The electrically modified deposition of RP3HT (Δ*m* > 0 for *Usb* = 30 V in Figure 5a) is postulated to be related with the precipitation of oxidized aggregates [36,41], which should also increase the crystallinity degree and modify charge transport properties of the deposited film. To examine this issue, we performed an additional experiment for pristine regioregular RP3HT. The RP3HT thin film was fabricated on scale-up substrates with 12 sets of ITO finger electrodes (supplied by Ossila) (Figure 6). The finger electrodes were grounded, whereas the sequence of *Usb*(*t*) 30 V, −30 V, and 0 V was applied to the barrier in the course of horizontal-dip coating of the RP3HT film. After film drying, the current–voltage characteristics of the fabricated electric circuits were measured in argon atmosphere. It is clearly visible that the conductance of the fabricated circuits is greatly increased (about 40 times) for the polymer films sections formed at *Usb* = 30 V as compared to −30 V and 0 V (Figure 6).

The other findings of Figure 5a,b and Figure 6 are related to the specific properties of the studied system. First, short exposures to small potential differences *Usb* control the RP3HT content in the solution volume that forms the coated layer. This process occurs before the layer leaves the meniscus region entrained by the substrate, resulting in the macroscopic onset of phase separation. Second, the nonzero electrical effects on the composite morphology, equivalent to additional RP3HT loading on the substrate (Δ*m* > 0), are observed only for the substrate positively polarized with respect to the barrier (Figure 5a and Figure 6, positive *Usb*). To resolve this asymmetry, we recall the coating flow fields between the stationary barrier and the moving substrate implied by Landau–Levich theory [59,60]. According to the fluid streamlines (Figure 5c–e), the coating solution layer splits into two around a stagnation point, and only the lower part is entrained by the substrate, while the upper part returns along the barrier to the reservoir in the meniscus. This situation introduces asymmetry to the otherwise symmetric effect of solubility controlled by the nearby electrode (positively polarized substrate or barrier).

## 4. Conclusions

In summary, our work has demonstrated that the morphology of three different polythiophene composites can be electrically controlled during dip coating onto a rapidly drawn horizontal solid substrate, both rigid and flexible. This methodology for morphology control should be broadly applicable to all meniscus-guided coating techniques and compatible with roll-to-roll processes for large-area organic electronics. The key concept in our approach, rarely previously employed, is the temporal variation in the coating process, which introduces morphology changes along the deposited composite. 

In our work, rapid control of the meniscus-guided coating was introduced through variation in the electric potential difference applied across the meniscus. We related the demonstrated morphology control to the voltage-induced doping and doping-dependent solubility of conjugated polymers, equivalent to a modified semiconductor content that controls the morphology. Therefore, the potential applications of our approach include, in addition to polythiophene composites, all conjugated polymer systems. 

The mechanism of voltage-controlled doping and solubility, when applied to pristine polythiophene solutions, results in an increased polythiophene deposition accompanied by highly improved charge transport properties of the deposited film. This suggests possible applications also for electrically modified horizontal-dip coating of pristine semiconductor films. 

## 5. Patents

The Jagiellonian University patented (PL 230245, EP 3519153, US 10,814,531) the method and equipment described in this manuscript, of which K.A., J.R., M.M.M. and A.B. are inventors.

## Data Availability

The data presented in this study are available on request from the corresponding author.

## Supplementary Materials

The following supporting information can be downloaded at: https://www.mdpi.com/article/10.3390/ma15062219/s1, Figure S1. AFM-based control of the barrier (B) height and tilt in relation to the substrate; Figure S2. Comparison of (a,c) AFM (height) and (b,d) ToF–SIMS chemical images of RP3HT/PEG-PCL composites identifying the elevated domains rich in RP3HT and the depressed domains rich in PEG-PCL; Figure S3. Comparison of AFM (height) (1st row) and ToF–SIMS chemical (2nd row) images of RP3HT/PEG-PCL (50:50) composites directly identifying the depressed domains rich in PEG-PCL and indirectly identifying the elevated domains rich in RP3HT; Figure S4. Electrically switched changes between film morphologies of various conductive polymer composites; Figure S5. Comparison of AFM (height) (1st row), optical (2nd row), and fluorescence (3rd row) images of RP3HT/PMMA (55:45) composites directly identifying the depressed domains rich in fluorescent RP3HT and indirectly identifying the elevated domains rich in PMMA; Figure S6. Comparison of ToF–SIMS mass signals characteristic for thiophene normalized to sum of all signals, recorded for the RP3HT/PEG-PCL (50:50) composite coated under voltage-off conditions prior to (R1) and after (R3) the voltage pulse. (a) Normalized signals C_6_HS^−^ (m/z = 104,9817), C_4_HS^−^ (m/z = 80,9817), and C_4_S^−^ (m/z = 79,9643). (b) The ratio R3/R1 of the signals recorded after (R3) and prior to (R1) the voltage pulse with their weighted average; Figure S7. (a) Ellipsometry spectra of the extinction coefficient determined along a pristine RP3HT layer H-dip-coated from chlorobenzene compared with (b) the absorption coefficient of RP3HT spin-coated from chlorobenzene and the theoretical prediction for the lower energy spectrum fraction characteristic of aggregate absorption. References [61,62] are cited in the Supplementary Materials.
