# Supplementary material for: Electrically Switchable Film Structure of Conjugated Polymer Composites
Source: Materials (Basel). 2022 Mar 17;15(6):2219. doi: 10.3390/ma15062219 (PMC8951423; doi:10.3390/ma15062219)
Supplement: Supplementary file 1 [file materials-15-02219-s001.zip › materials-1624034 supplementary.pdf]

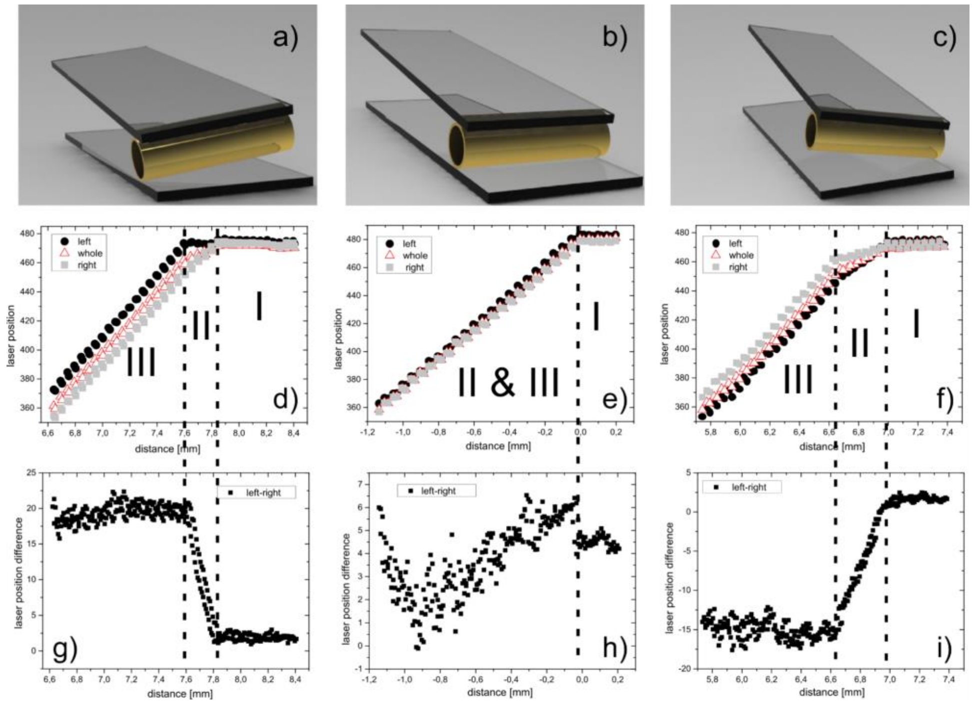

Figure S1. AFM-based control of the barrier (B) height and tilt in relation to the substrate (S), tracing the mean position of the whole linear laser beam reflected by the support of the cylindrical barrier, as well as the positions of the beam reflected from the right and left ends of the barrier's support. **a-c**, Sketches of various positions of the barrier in relation to the substrate; **d-f**, plots of the laser spot location *vs.* the height of the barrier holder above the substrate; **g-i**, plots of the difference in the laser spot location *vs.* the height of the barrier holder above the substrate. The cases of a higher positioning of the left (**a, d, g**) or right (**c, f, i**) end of the barrier as well as parallel barrier positioning (**b, e, h**) in relation to the substrate are presented. The positioning stages are marked: (I) no contact of the barrier with the substrate; contact with one (II) or the other (III) end of the barrier.

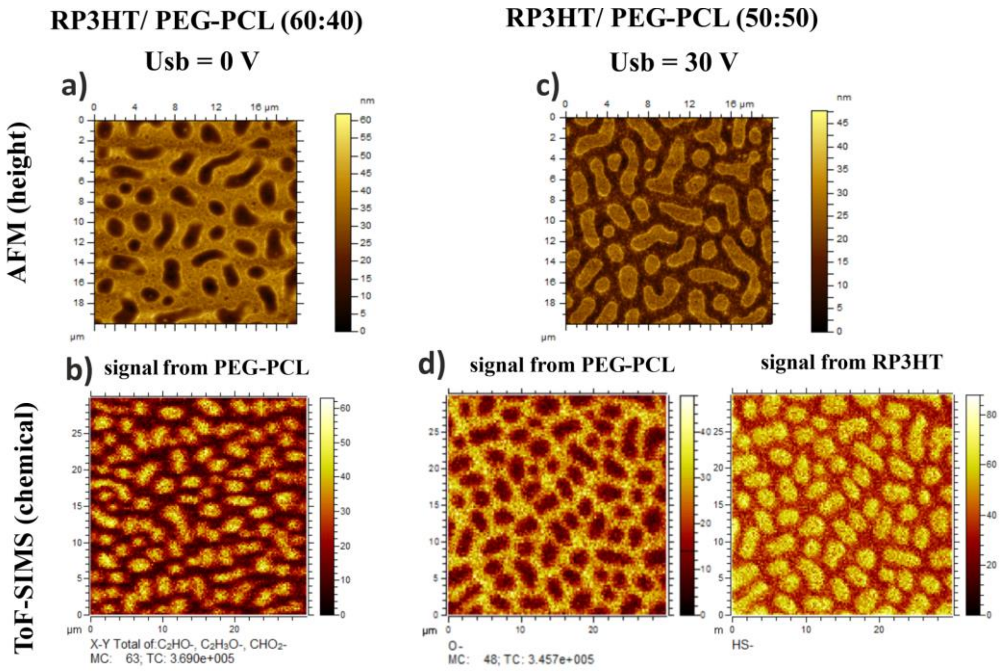

Figure S2. Comparison of (a, c) AFM (height) and (b, d) ToF-SIMS chemical images of RP3HT/PEG-PCL composites identifying the elevated domains rich in RP3HT and the depressed domains rich in PEG-PCL. Surfaces of the pair of composites with phase-inverted droplet-like lateral morphologies. ToF-SIMS chemical images using different secondary ions as the signals characteristic of PEG-PCL (total of C_2HO^-, $C_2H_2O^-$, and CHO_2^- or O^-) and RP3HT (HS^-).

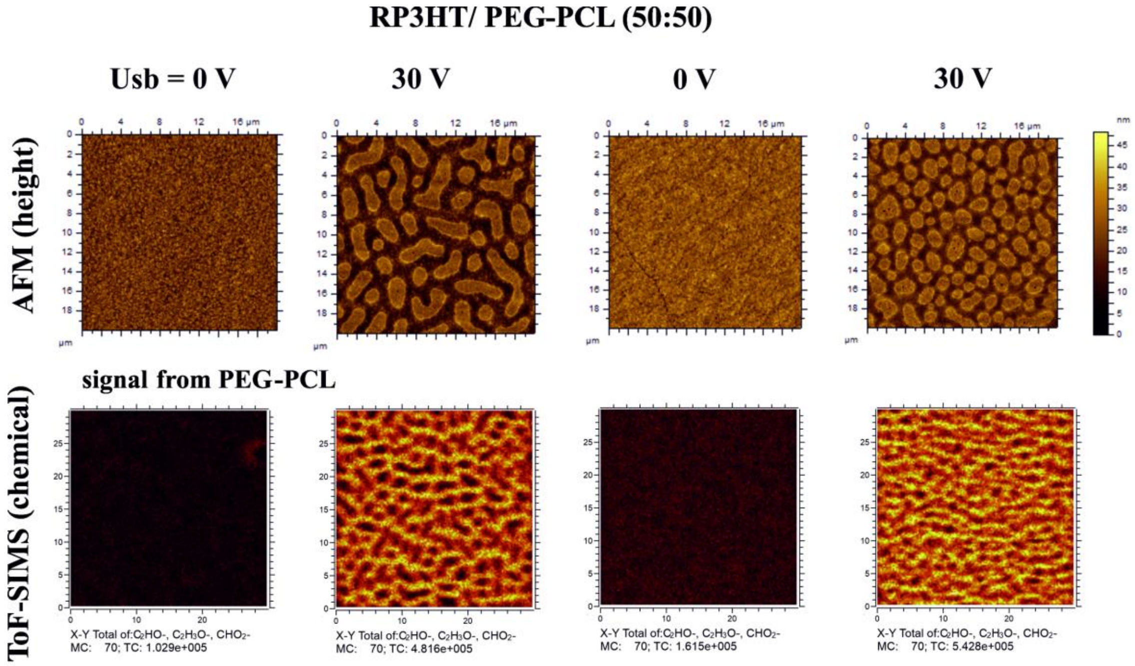

Figure S3. Comparison of AFM (height) (1st row) and ToF-SIMS chemical (2nd row) images of RP3HT/PEG-PCL (50:50) composites directly identifying the depressed domains rich in PEG-PCL and indirectly identifying the elevated domains rich in RP3HT. The surfaces of the composite show the E-field-induced changes from the lamellar structure (see the 1st and 3rd columns, with the phase rich in PEG-PCL absent at the surface) into the

lateral morphology (see the 2nd and 4th columns, with both phases present). The ToF-SIMS chemical maps are based on the signals characteristic of PEG-PCL (total of C_2HO^-, $C_2H_2O^-$, and CHO_2^-).

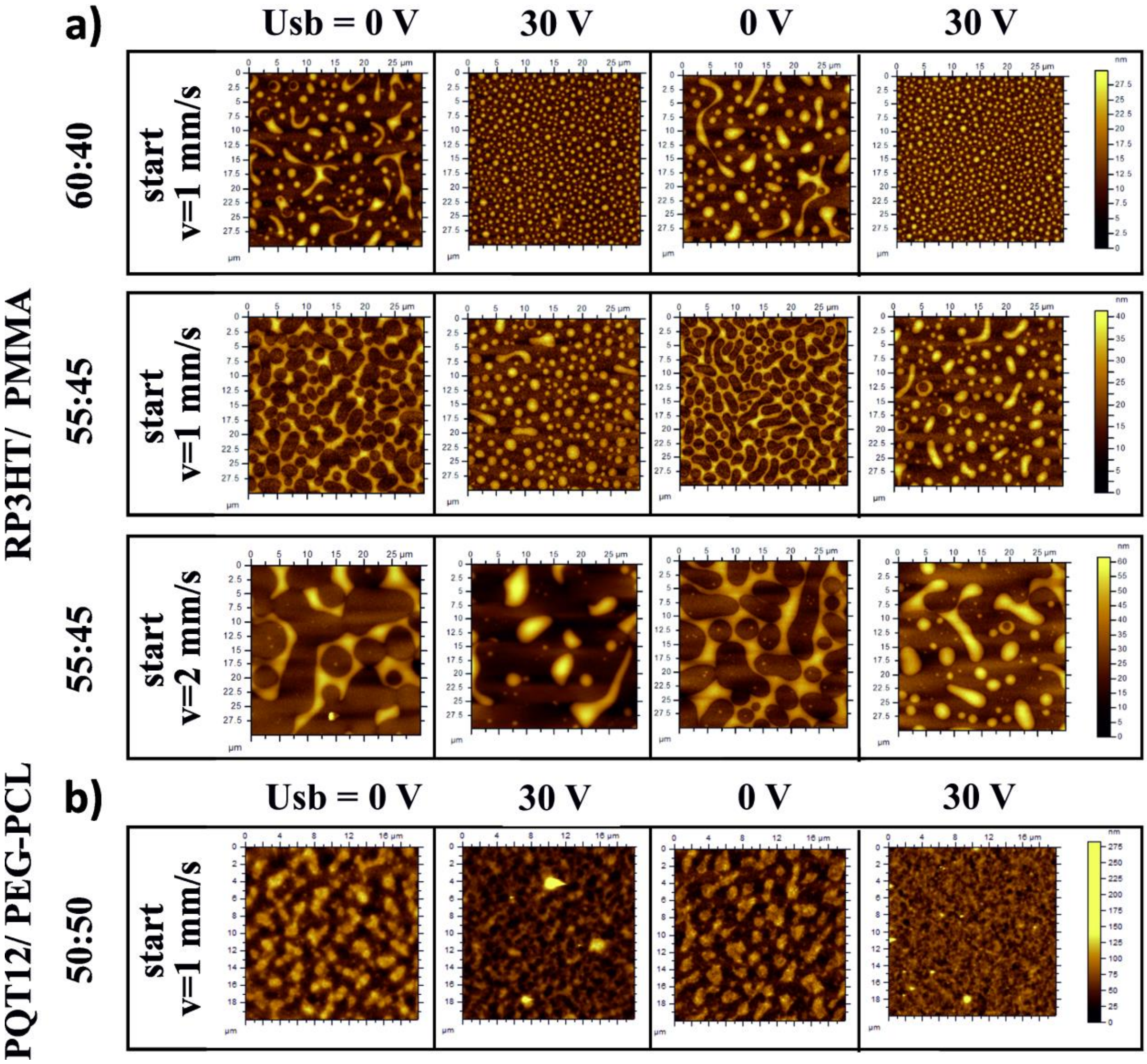

Figure S4. Electrically switched changes between film morphologies of various conductive polymer composites. AFM height images of the composites (**a**) RP3HT/PMMA and (**b**) PQT12/PEG-PCL H-dip coated with the voltage *Usb* alternating between 0 V and 30 V (columns). Rows group the results for different weight ratios of components or for different coating speeds *v*. The data reveal for both phase-separated blends the distinct film structures that are controlled by the E-field in a reversible and repeatable manner. In particular, electrically induced changes between the phase-inverted droplet-like lateral structures are demonstrated for two coating speeds (cf. the 2rd and 3rd rows). AFM height images in (a) reflect elevated domains rich in PMMA and depressed regions rich in RP3HT (cf. Fig. S5).

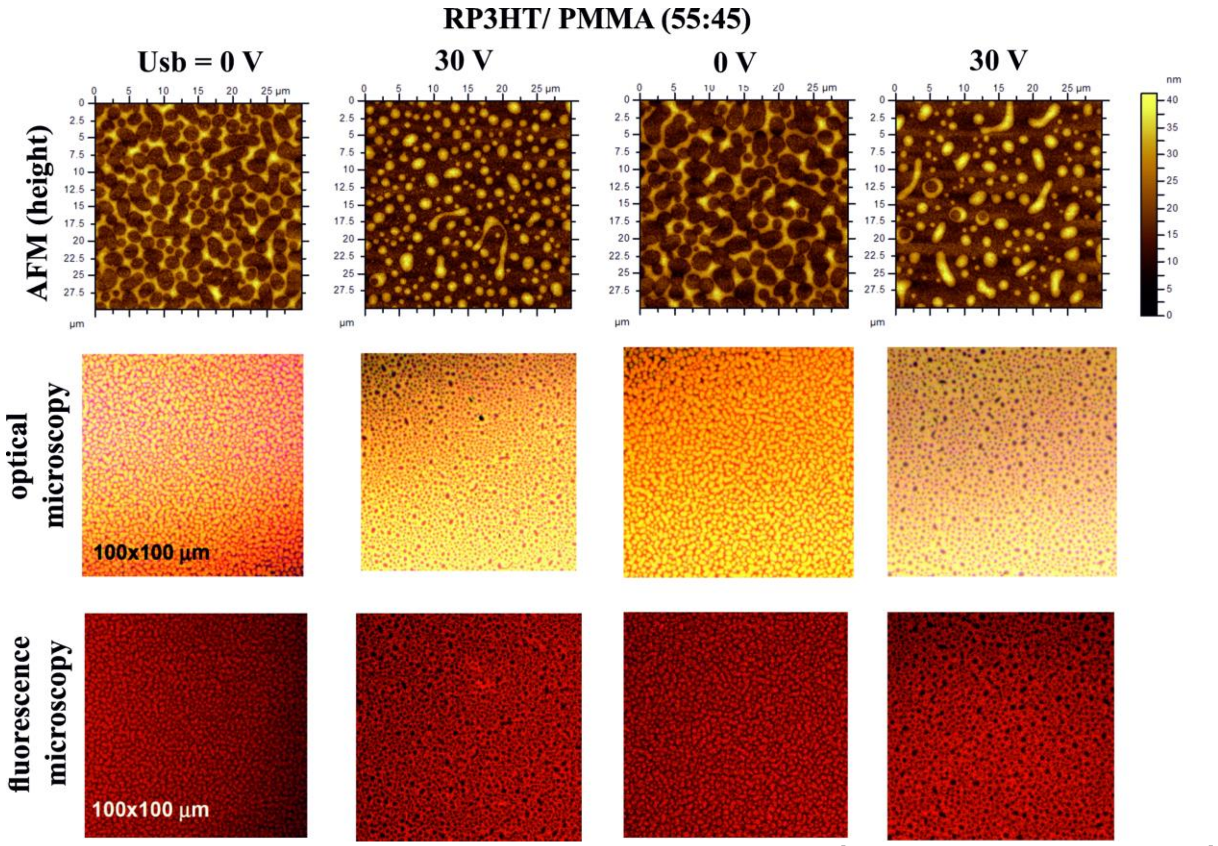

Figure S5. Comparison of AFM (height) (1st row), optical (2nd row) and fluorescence (3rd row) images of RP3HT/PMMA (55:45) composites directly identifying the depressed domains rich in fluorescent RP3HT and indirectly identifying the elevated domains rich in PMMA. The surfaces of the composite show the E-field-induced changes between the phase-inverted droplet-like lateral morphologies (the 1st and 3rd columns vs. the 2nd and 4th columns).

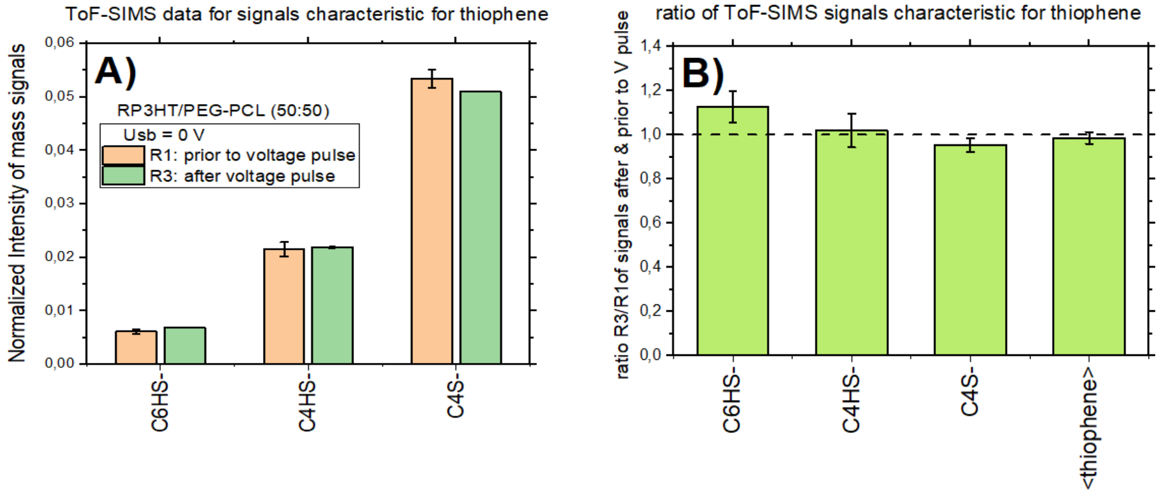

Figure S6. Comparison of ToF-SIMS mass signals characteristic for thiophene rings normalized to sum of all signals, recorded for the RP3HT/PEG-PCL (50:50) composite coated at voltage-off conditions prior to (R1) and after (R3) the voltage pulse (cf. the 1st and the 3rd column in Figs. 2 and S3). (a) Normalized signals C_6HS^- (m/z = 104,98), C_4HS^- (m/z = 80,98) and C_4S^- (m/z = 79,96). (b) The ratio R3/R1 of the signals recorded after (R3) and prior to (R1) the voltage pulse with their weighted average. Changes in spectroscopic

signals characteristic for thiophene rings are considered as a token of chemical degradation of polythiophene [46].

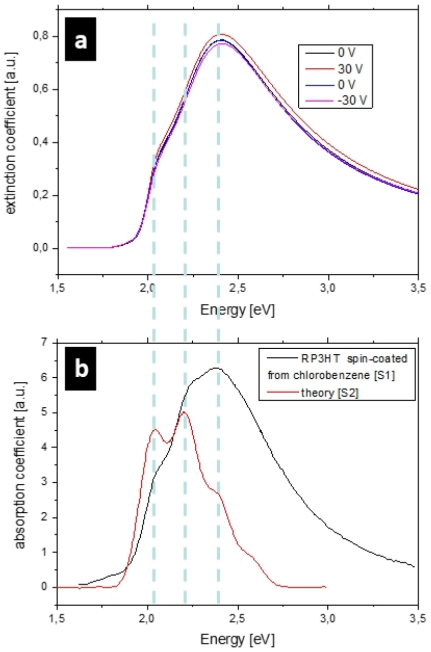

Figure S7. (a) Ellipsometry spectra of the extinction coefficient determined along a pristine RP3HT layer H-dip coated from chlorobenzene compared with (b) the absorption coefficient of RP3HT spin-coated from chlorobenzene [61] and the theoretical prediction for the lower energy spectrum fraction characteristic of aggregate absorption [62]. The determined spectrum (a) reveals the same features (marked by dashed lines) as the spectrum reported earlier [61], with the absorption peak at 2.39 eV and shoulders at 2.23 and 2.05 eV [61]. The shoulders correspond to vibronic bands with 0-1 and 0-0 transitions, respectively [62], formed upon aggregation [62].
